# Supplementary material for: Ag2O and NiO Decorated CuFe2O4 with Enhanced Photocatalytic Performance to Improve the Degradation Efficiency of Methylene Blue
Source: Materials (Basel). 2020 Oct 25;13(21):4760. doi: 10.3390/ma13214760 (PMC7662239; doi:10.3390/ma13214760)
Supplement: Supplementary file 1 [file materials-13-04760-s001.pdf]

materials

Supplementary Information

Ag₂O and NiO Decorated CuFe₂O₄ with Enhanced Photocatalytic Performance to Improve the Degradation Efficiency of Methylene Blue

Lu Liu [1,2,*], Nan Hu [1,2,*], Yonglei An [3,4], Xingyuan Du [3,4], Xiao Zhang [1], Yan Li [1], Yan Zeng [1] and Zheng Cui [5]

1 School of Energy and Power Engineering, Changchun Institute of Technology, Changchun 130012, China; zhangx049@nenu.edu.cn (X.Z.); moon471285743@126.com (Y.L.); zengyan@ccit.edu.cn (Y.Z.)
2 Jilin Province S&T Innovation Center for Physical Simulation and Security of Water Resources and Electric Power Engineering, Changchun Institute of Technology, Changchun 130012, China
3 Key Laboratory of Groundwater Resources and Environment, Jilin University, Ministry of Education, Changchun 130021, China; anyonglei85@jlu.edu.cn (Y.A.); duxy19@mails.jlu.edu.cn (X.D.)
4 Jilin Provincial Key Laboratory of Water Resources and Environment, Jilin University, Changchun 130021, China
5 State Key Laboratory of Superhard Materials, College of Physics, Jilin University, Changchun 130012, China; cuizheng18@mails.jlu.edu.cn
* Correspondence: liulu@ccit.edu.cn (L.L.); hunan@ccit.edu.cn (N.H.)

Received: 2 September 2020; Accepted: 22 October 2020; Published: 25 October 2020

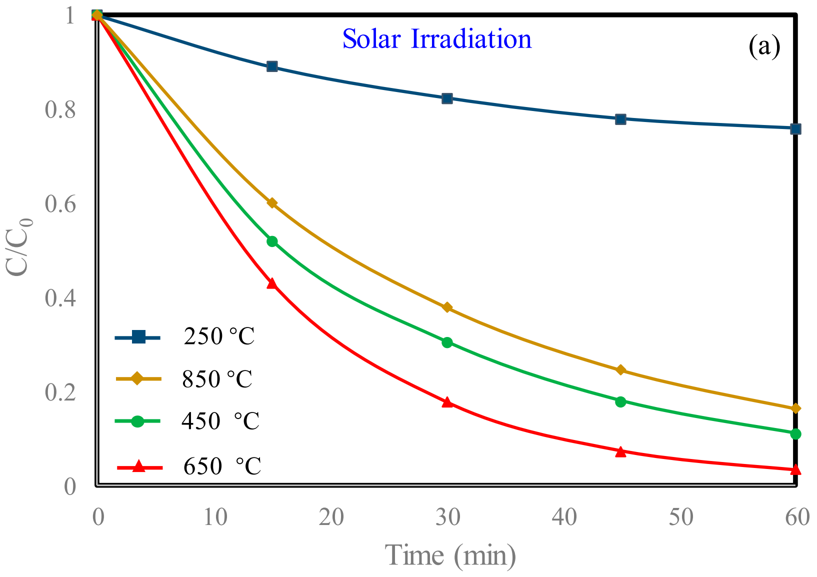

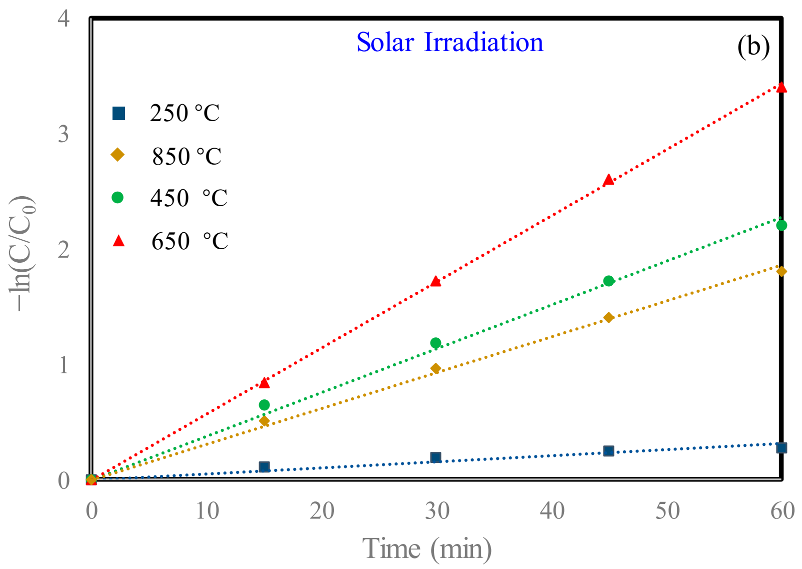

Figure S1. Dependance of photodegradation of MB dye on sintering temperature in Muffle furnace. (**a**) Degradation of MB in samples sintered at different temperatures; (**b**) The pseudo-first-order reaction kinetics for MB degradation with different samples.

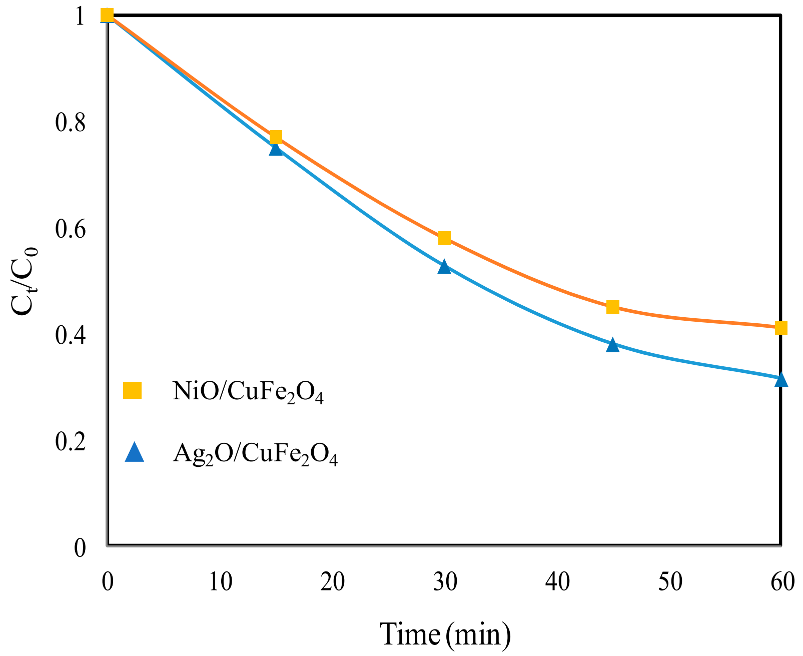

Figure S2. Degradation of MB with $Ni/CuFe_2O_4$ and $Ag_2O/CuFe_2O_4$ under simulated solar irradiation. Experimental conditions: H_2O_2 100 uL, Catalyst 10 mg. The degradation rates of MB by $Ni/CuFe_2O_4$ and $Ag_2O/CuFe_2O_4$ were 58.92% and 68.91% respectively, which are both higher than $CuFe_2O_4$(57.88%).

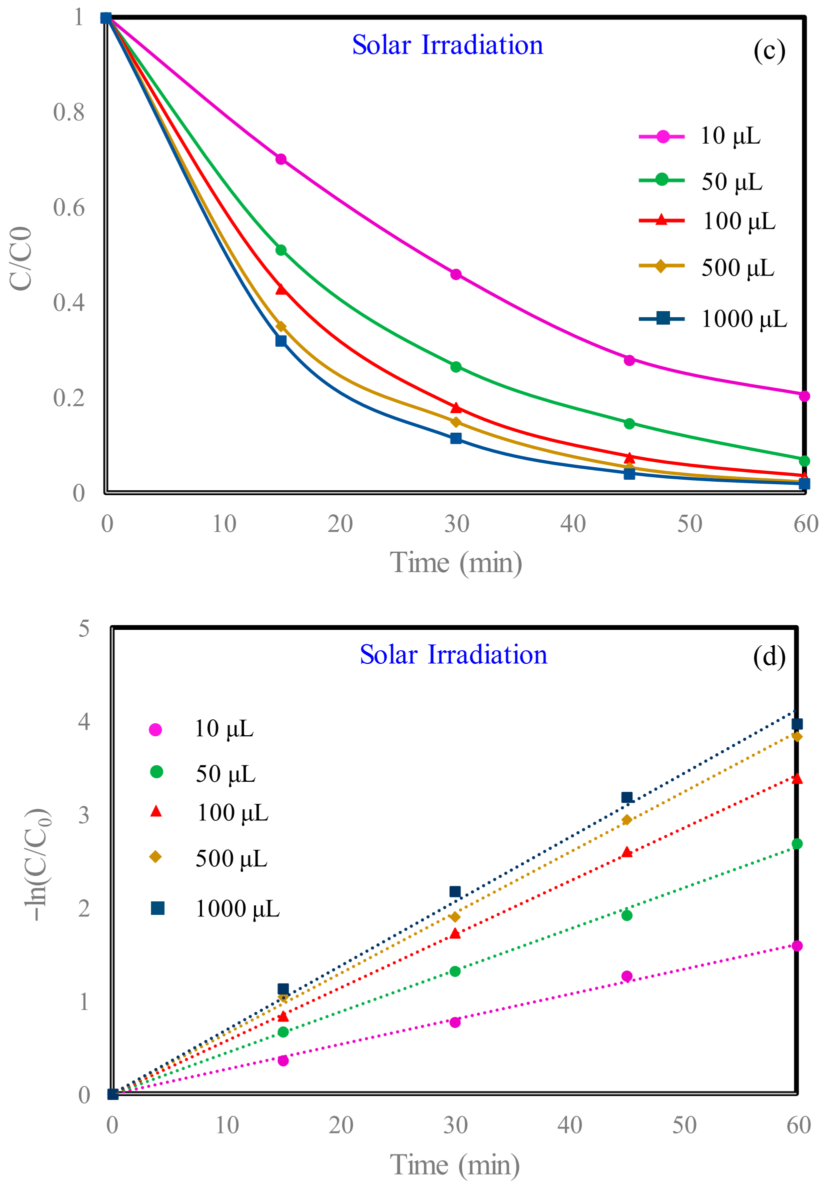

Figure S3. Effect of H_2O_2 dosage on the degradation rate of MB. (**a**) MB degradation with different H_2O_2 dosage; (**b**) The pseudo-first-order reaction kinetics for MB degradation with different H_2O_2 dosage.

Publisher's Note: MDPI stays neutral with regard to jurisdictional claims in published maps and institutional affiliations.

© 2020 by the authors. Licensee MDPI, Basel, Switzerland. This article is an open access article distributed under the terms and conditions of the Creative Commons Attribution (CC BY) license (http://creativecommons.org/licenses/by/4.0/).
